# Supplementary material for: Immune Cells Are Differentially Modulated in the Heart and the Kidney during the Development of Cardiorenal Syndrome 3
Source: Cells. 2023 Feb 13;12(4):605. doi: 10.3390/cells12040605 (PMC9953884; doi:10.3390/cells12040605)
Supplement: Supplementary file 1 [file cells-12-00605-s001.zip › cells-2171550-supplementary.pdf]

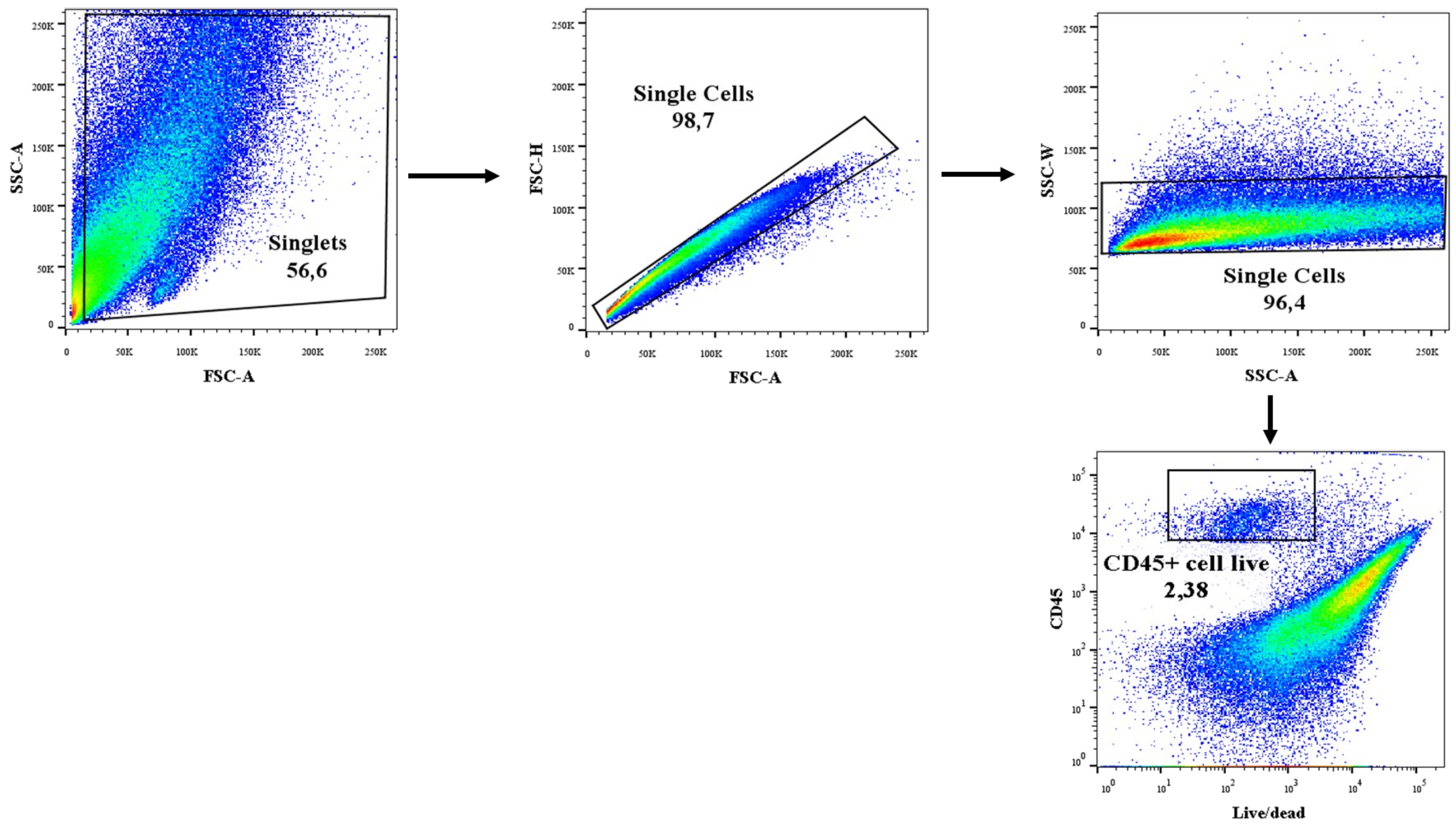

Supplementary Figure S1: Gate strategy used to exclude dead and doublets cells. Suspended cells from samples of the kidney and heart were gated on FSC-A and SSC-A, followed by FSC-H vs FSC-A and SSC-W vs SSC-A. After, based on the use of Live&dead kit and CD45 we identified live immune cells for the ones that were negative for live&dead and positive for CD45.
